# Functional Evaluation of *Plasmodium* Export Signals in *Plasmodium berghei* Suggests Multiple Modes of Protein Export

**DOI:** 10.1371/journal.pone.0010227

**Published:** 2010-04-19

**Authors:** Puran Singh Sijwali, Philip J. Rosenthal

**Affiliations:** 1 Centre for Cellular and Molecular Biology, Hyderabad, India; 2 Department of Medicine, San Francisco General Hospital, University of California San Francisco, San Francisco, California, United States of America; New York University School of Medicine, United States of America

## Abstract

The erythrocytic stage development of malaria parasites occurs within the parasitophorous vacuole inside the infected-erythrocytes, and requires transport of several parasite-encoded proteins across the parasitophorous vacuole to several locations, including the cytosol and membrane of the infected cell. These proteins are called exported proteins; and a large number of such proteins have been predicted for *Plasmodium falciparum* based on the presence of an N-terminal motif known as the *Plasmodium* export element (PEXEL) or vacuolar transport signal (VTS), which has been shown to mediate export. The majority of exported proteins contain one or more transmembrane domains at the C-terminus and one of three types of N-terminus domain architectures. (1) The majority, including the knob-associated histidine rich protein (KAHRP), contain a signal/hydrophobic sequence preceding the PEXEL/VTS motif. (2) Other exported proteins, including the *P. berghei* variant antigen family bir and the *P. falciparum* skeleton binding protein-1, do not appear to contain a PEXEL/VTS motif. (3) The *P. falciparum* erythrocyte membrane protein-1 (PfEMP1) family lacks a signal/hydrophobic sequence before the motif. These different domain architectures suggest the presence of multiple export pathways in malaria parasites. To determine if export pathways are conserved in plasmodia and to develop an experimental system for studying these processes, we investigated export of GFP fused with N- and C-terminus putative export domains in the rodent malaria parasite *P. berghei*. Export was dependent on specific N- and C-terminal domains. Constructs with a KAHRP-like or bir N-terminus, but not the PfEMP1 N-terminus, exported GFP into the erythrocyte. The C-terminus of a *P. falciparum* variant antigen rifin prevented GFP export by the KAHRP-like N-terminus. In contrast, GFP chimeras containing KAHRP-like N-termini and the PfEMP1 C-terminus were exported to the surface of erythrocytes. Taken together, these results suggest that proteins with KAHRP-like architecture follow a common export pathway, but that PfEMP1s utilize an alternative pathway. Functional validation of common putative export domains of malaria parasites in *P. berghei* provides an alternative and simpler system to investigate export mechanisms.

## Introduction

The development of erythrocytic malaria parasites is accompanied by transport of parasite-encoded proteins beyond the parasitophorous vacuole membrane (PVM) to multiple locations, including the cytoplasm and membrane of the infected-erythrocyte. These proteins confer remarkably altered properties to infected erythrocytes, including increased permeability to a variety of substances, increased rigidity, and adherence to endothelial cells [1]. A key family of *P. falciparum* exported proteins is collectively termed *P. falciparum* erythrocyte membrane protein-1 (PfEMP1). These proteins are transported to the surface of erythrocytes to mediate cytoadherence to vascular endothelial cells, a key pathogenic mechanism of *P. falciparum* [2]. Members of the PfEMP1family are diverse in sequence, and only one member is predominantly expressed in a given developmental cycle, thus allowing antigenic variation to evade the host immune response [3]. PfEMP1 is anchored on the surface of parasitized erythrocytes in electron dense knobs through interactions with erythrocyte cytoskeleton proteins and parasite encoded proteins, including the knob-associated histidine-rich protein (KAHRP) [2]. Some other characterized exported proteins include rifins and stevors, two other variant antigen families of unknown function [4], [5], [6], skeleton binding protein-1 (SBP1) [7], ring-exported protein-2 (REX2) [8], and surfins [9]. SBP1 and RXP2 are associated with the Maurer's cleft [7], [8], and SBP1 also plays a key role in the transport of PfEMP1 [10], [11]. Surfins are polymorphic antigens localized to the PVM and the surface of infected erythrocytes, and have unknown function [9]. Other malaria parasites also have rifin-like variant antigen proteins, including the bir protein family in *P. berghei* [12].

A five amino acid sequence (RxLxQ/E) termed the *Plasmodium* Export Element (PEXEL) or Vacuolar Targeting Signal (VTS) has been identified in the amino terminus of the majority of known exported proteins of *P. falciparum* [13], [14]. N-termini containing the PEXEL/VTS motif of several exported proteins, including PfEMP1, have been shown to export GFP in *P. falciparum*-infected erythrocytes [13], [14]. Also, the PEXEL/VTS motifs of some predicted exported proteins of *P. vivax* and *P. gallinaceum* exported GFP in *P. falciparum*-infected erythrocytes [13]. Bioinformatic analyses identified several proteins with PEXEL/VTS motifs in multiple *Plasmodium* species, including the mouse model parasite *P. berghei* [15]. The PEXEL motif has been shown to be processed in the endoplasmic reticulum, and this cleavage appears to be a prerequisite for export because mutations in this motif abrogate both processing and export of PEXEL-containing proteins [16], [17]. Recently, plasmepsin V, an endoplasmic reticulum resident aspartic protease, has been shown to process the PEXEL motif [18], [19]. Additionally, a proposed translocon for protein export has been recently discovered in the parasitophorous vacuole membrane (PVM) of *P. falciparum* [20].

Several exported proteins, including SBP1, surfins, and the *P. berghei* variant antigen family bir lack the PEXEL/VTS motif [21], and export signals for such proteins have not been identified. The majority of exported proteins contain one or more transmembrane domains at the C-terminus, and share three types of N-terminus domain architectures [21]. (1) The N-termini of the majority of exported proteins (here termed Type 1), including KAHRP, rifins, and stevors, contain a hydrophobic/signal sequence preceding the PEXEL/VTS motif. (2) Several other known exported proteins (Type 2), including SBP-1, REX2 and bir proteins do not contain the PEXEL/VTS motif. (3) Lastly, PfEMP1 proteins do not have a signal/hydrophobic sequence before the PEXEL/VTS (Type 3). Several type-1 N-termini, including that of KAHRP, which contains a signal/hydrophobic sequence followed by the PEXEL/VTS motif, have been shown to be sufficient for export of reporter proteins into the *P. falciparum*-infected erythrocyte [13], [14]. For Type-2 proteins like SBP1 and RXP2, both N and C-termini have been shown to be required for export [8], [22]. For PfEMP1s, which represent Type 3 proteins, different lengths of the N-terminus together with the C-terminus appear to be required for export [13], [14], [23].

Different domain architectures of plasmodial exported proteins suggest multiple export pathways in malaria parasites [24]. Characterization of these export pathways may facilitate the identification of novel drug or vaccine targets. As the rodent parasite *P. berghei* offers a simple animal model and much more efficient genetic manipulation than *P. falciparum* [25], [26], it may facilitate characterization of plasmodial protein export, especially since it was recently shown that a PEXEL/VTS motif allowed GFP export into the *P. berghei*-infected erythrocyte [27]. To determine if malaria parasites have evolved multiple mechanisms for exporting different types of proteins, we transfected *P. berghei* with plasmids expressing GFP fused with portions of a number of exported proteins representing the three domain architectures described above and evaluated transport of chimeras in infected erythrocytes.

## Results

### Construction of GFP chimeras

**Figure 1:**
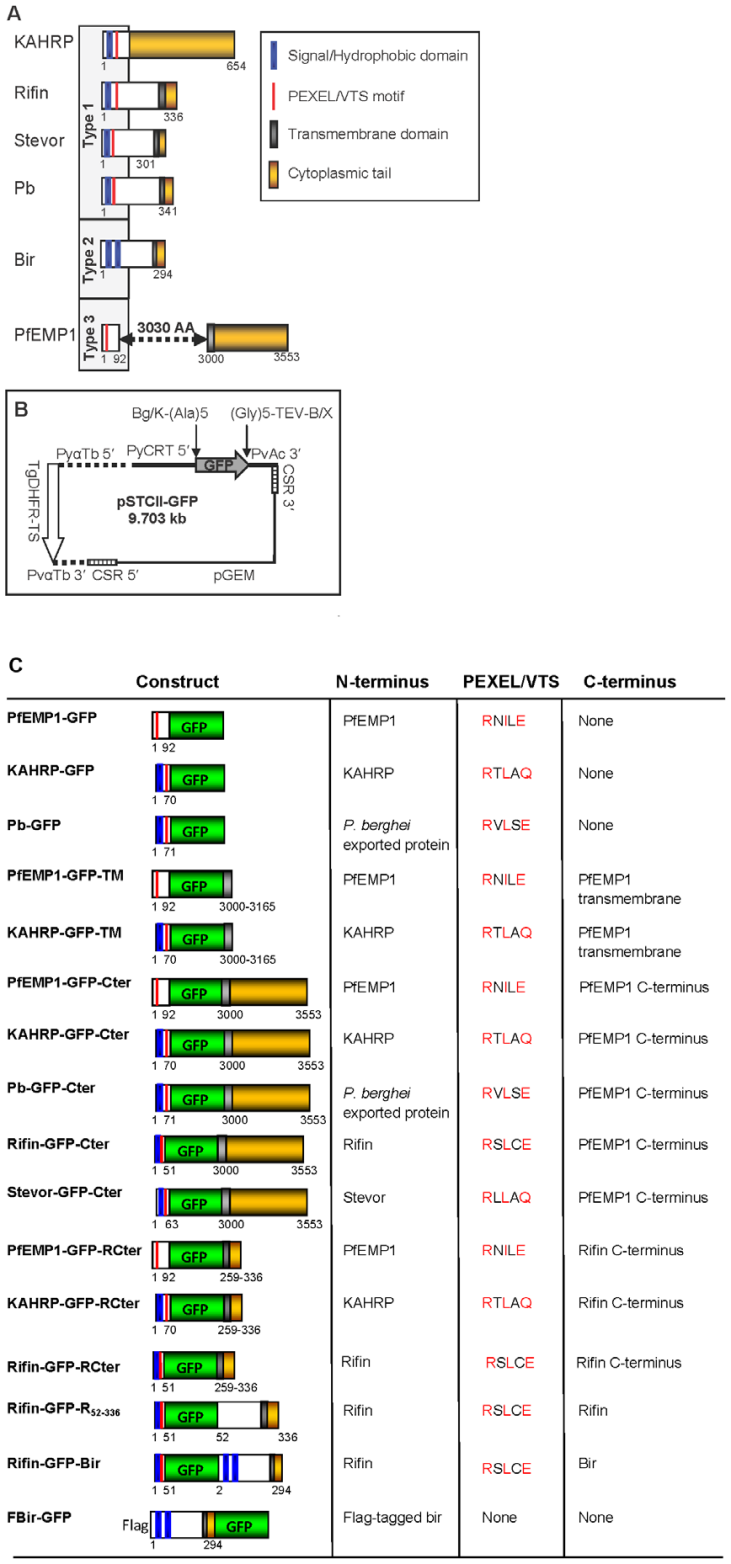
Schematics of exported proteins and transfection plasmid. (**A**) The three major types of architectures seen among exported proteins are shown with amino acid numbers indicated. The Type 1 proteins, represented by KAHRP, rifin, stevor, and a predicted *P. berghei* exported protein, contain a signal/hydrophobic sequence preceding the PEXEL/VTS motif. The Type 2 proteins, represented by the *P. berghei* variant antigen bir, lack the PEXEL/VTS motif. The Type 3 proteins, including the PfEMP1 family, lack a signal/hydrophobic sequence before the PEXEL/VTS motif. (**B**) The transfection plasmid pSTCII-GFP expresses GFP under the control of the *P. yoelii* chloroquine resistance transporter (PyCRT 5′) upstream and the *P. vivax* actin (PvAc 3′) downstream untranslated regions. It has pGEM backbone for replication in *E. coli*, and the pyrimethamine resistant *Toxoplasma gondii* dihydrofolate reductase-thymidylate synthase gene (TgDHFR-TS) under the control of the *P. yoelii* α tubulin (PyαTb 5′) upstream and the *P. vivax* α tubulin (PvαTb 3′) downstream untranslated regions for selection of transfected parasites. (**C**) Schematic of GFP reporter constructs utilized in this report showing different regions of exported proteins. GFP is green and the remainder of the color scheme is as defined in panel A.

To determine if malaria parasites have evolved a single or multiple mechanisms for exporting proteins with different domain architectures and to identify functionally conserved export elements, we constructed GFP chimeras containing N-termini of proteins representing the three domain architectures of plasmodial exported proteins (Fig. 1). Representative Type 1 proteins, which include the majority of exported proteins with a signal/hydrophobic sequence preceding the PEXEL/VTS motif, were KAHRP (PFB0100c), rifin (PFA0745w), stevor (PFC1105w), and a predicted *P. berghei* exported protein (PB402722.00.0). The Type 2 proteins, in which the PEXEL/VTS motif is absent, were represented by a bir protein. The Type 3 domain architecture, which lacks a signal/hydrophobic sequence before the PEXEL/VTS motif, was represented by a PfEMP1 protein (PFD1235w). Additionally, to determine the effect of the C-terminus on export, GFP chimeras containing the C-termini of PfEMP1, rifin, and bir were constructed (Fig. 1). GFP chimeras were transfected into *P. berghei* using the plasmid pSTCII-GFP (Fig. 1), and live infected-erythrocytes were examined using an epifluorescence microscope to determine whether GFP was retained in the parasite or exported to the infected erythrocyte.

### N-termini with a signal/hydrophobic sequence preceding the PEXEL/VTS motif exported GFP

**Figure 2:**
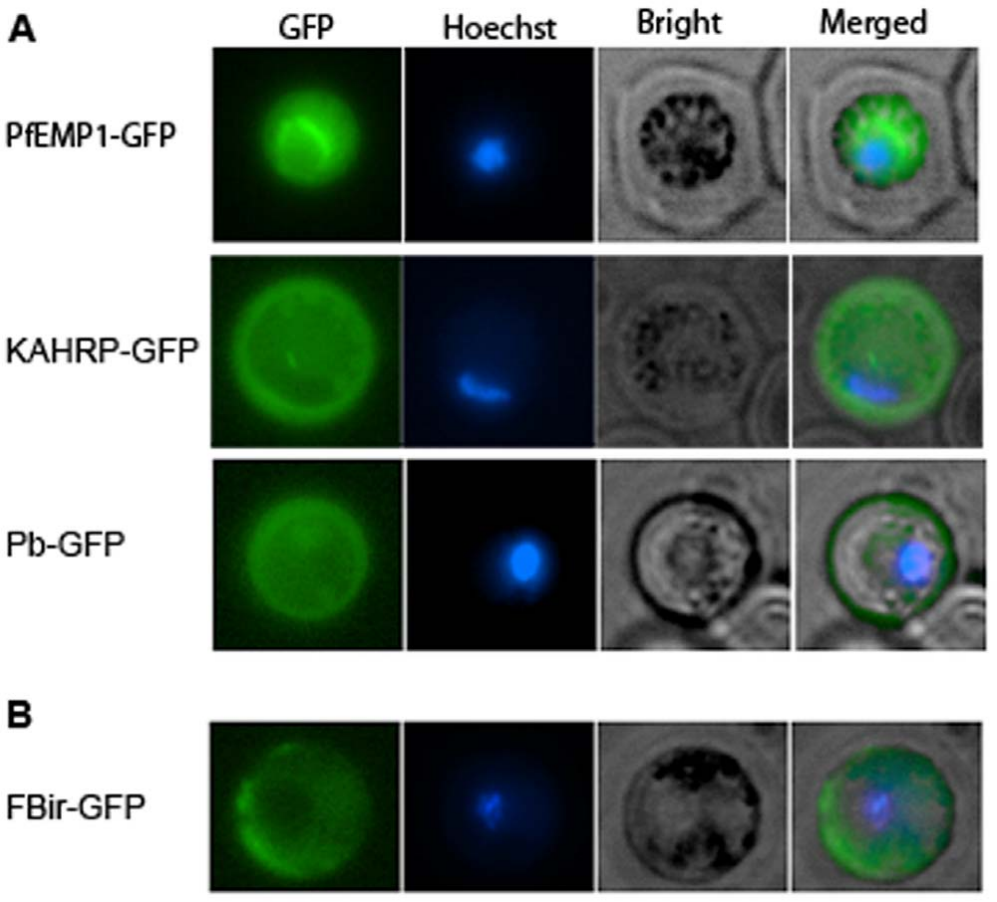
N-termini with a signal/hydrophobic sequence preceding the PEXEL/VTS motif exported GFP. (**A**) GFP reporters with the N-terminus of PfEMP1 (PfEMP1-GFP), KAHRP (KAHRP-GFP), and a predicted *P. berghei* exported protein (Pb-GFP) were expressed in *P. berghei*, and live parasites were stained with the DNA labeling dye Hoechst and viewed under an epifluorescence microscope. The expression of GFP, staining of the parasite nucleus (Hoechst), bright field images, and merged images are shown for representative trophozoite-stage parasites. (**B**) A GFP chimera of a flag-tagged *P. berghei* variant antigen bir (Fbir-GFP), which lacks the PEXEL/VTS motif, was expressed in *P. berghei*, and live trophozoite-stage parasites were imaged as described above.

To investigate if the PEXEL/VTS motif is functional in *P. berghei*, parasites were transfected with plasmids encoding GFP chimeras containing the N-terminus of PfEMP1 (PfEMP1-GFP), KAHRP (KHARP-GFP) and a predicted *P. berghei* exported protein (Pb-GFP); and the localization of GFP was examined in live infected cells. KAHRP-GFP and Pb-GFP expressing cells showed fluorescence in the erythrocyte cytosol, indicating that GFP was exported into erythrocytes (Fig. 2A). In PfEMP1-GFP expressing cells GFP was confined to the parasite cytosol, indicating that GFP was not exported into erythrocytes (Fig. 2A). Thus, the PEXEL/VTS motif is functional in *P. berghei*, mediating transport to the erythrocyte, but the motif functions only with an upstream signal/hydrophobic sequence.

### PEXEL/VTS motif independent export is present in *P. berghei*

To determine if a PEXEL/VTS independent export pathway is present in *P. berghei*, a GFP reporter containing a flag-tagged bir protein sequence (PB200037.00.0) at the N-terminus (FBir-GFP) was expressed in *P. berghei*. The FBir-GFP expressing cells showed fluorescence in the erythrocyte (Fig. 2B), indicating that GFP was exported, and thus providing evidence that a PEXEL/VTS independent pathway for transport from the parasite is conserved in Plasmodia.

### The C-terminus of rifin prevented export of GFP

**Figure 3:**
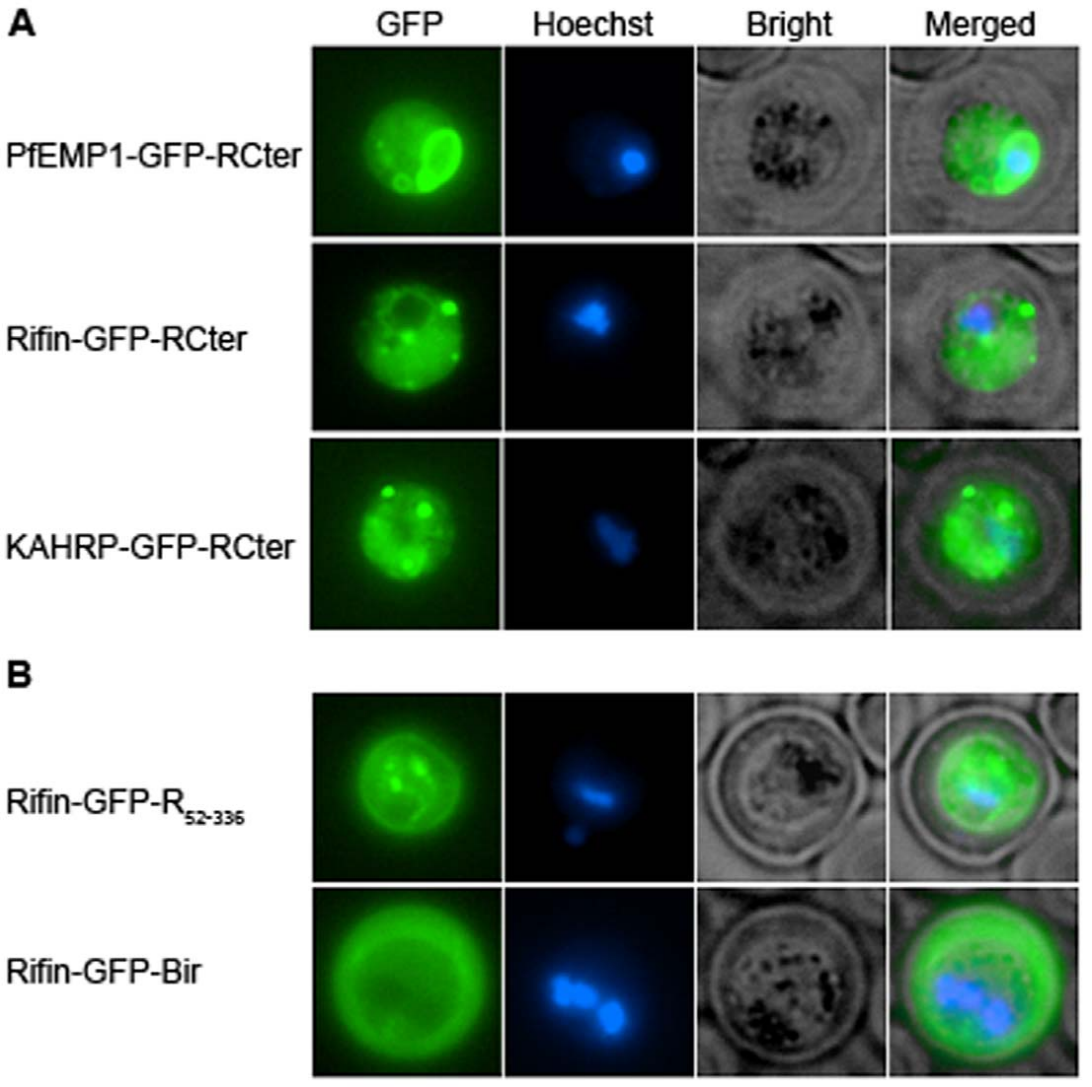
The C-terminus of rifin prevented GFP export. Trophozoite-stage parasites were imaged as described for Fig. 2. (**A**) Parasites expressing GFP chimeras containing the C-terminus of rifin at the carboxy terminus and the N-terminus of PfEMP1 (PfEMP1-GFP-RCter), rifin (Rifin-GFP-RCter) or KAHRP (KAHRP-GFP-RCter) at the N-terminus. (**B**) Parasites expressing GFP chimeras with the N-terminus of rifin upstream and the remaining region of rifin (Rifin-GFP-R_52-336_) or a bir protein (Rifin-GFP-Bir) downstream.

To determine if C-terminal regions of exported proteins affect export mediated by their cognate N-termini, GFP chimeras containing the C-terminus of rifin and the N-terminus of the same rifin protein (Rifin-GFP-RCter), the N-terminus of PfEMP1 (PfEMP1-GFP-RCter), or the N-terminus of KAHRP (KHARP-GFP-RCter) were expressed. As expected, since the PfEMP1 N-terminus did not export GFP, the PfEMP1-GFP-RCter protein was not transported into the erythrocyte (Fig. 3A). However, surprisingly, the C-terminus of rifin prevented GFP transport into the erythrocyte by the N-termini of KAHRP and rifin (Fig. 3A). To rule out the possibility that the C-terminus of rifin included in these reporter proteins lacked additional sequence necessary for export, a reporter containing the entire rifin sequence with GFP inserted between N-terminal amino acids 51–52 (Rifin-GFP-R_52-336_) was expressed in parasites. In parallel, another construct with the same length N-terminus of rifin followed by GFP and the bir sequence (Rifin-GFP-Bir) was also expressed in parasites. Rifin-GFP-R_52-336_ was not exported whereas Rifin-GFP-Bir was exported into the erythrocyte (Fig. 3B). Thus, at least in this case, the C-terminus of exported proteins carries destination specific information.

### The C-terminus of PfEMP1 did not affect GFP export

**Figure 4:**
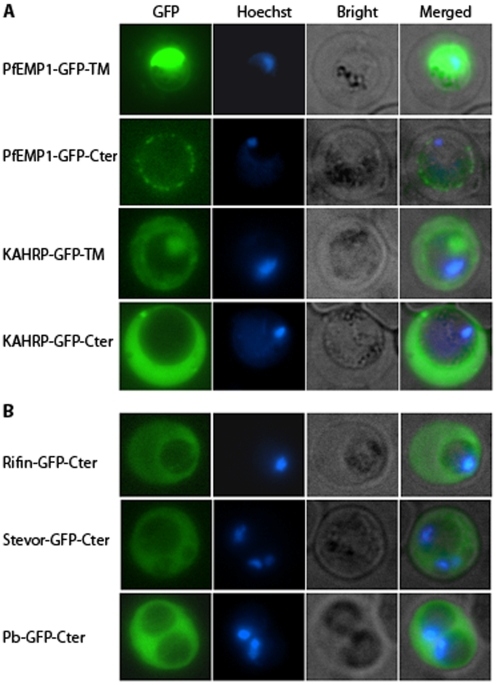
The C-terminus of PfEMP1 did not affect GFP export. Trophozoite-stage parasites were imaged as described for Fig. 2. (**A**) Parasites expressing GFP chimeras containing the N-terminus of PfEMP1 or KAHRP and the transmembrane domain or the C-terminus of PfEMP1 (PfEMP1-GFP-TM, KAHRP-GFP-TM, PfEMP1-GFP-Cter, KAHRP-GFP-Cter). (**B**) Parasites expressing GFP chimeras containing the C-terminus of PfEMP1 at the carboxy-terminus and N-terminus of rifin (Rifin-GFP-Cter), stevor (Stevor-GFP-Cter), and a predicted P. *berghei* exported protein (Pb-GFP-Cter) at the N-terminus were imaged.

The C-terminus of PfEMP1contains a transmembrane domain and a cytosolic domain designated the acidic terminal segment. To determine if these domains are required together with the PEXEL/VTS motif for the export of PfEMP1, GFP chimeras of the PfEMP1 N-terminus together with the transmembrane domain (PfEMP1-GFP-TM) or the entire C-terminus (PfEMP1-GFP-Cter) were expressed in parasites. Both PfEMP1-GFP-TM and PfEMP1-GFP-Cter expressing parasites showed fluorescence confined to the parasite; thus the PfEMP1 C-terminus region did not mediate transport to the erythrocyte (Fig. 4A). However, GFP chimeras containing the KAHRP N-terminus and the transmembrane domain (KHARP-GFP-TM) or the C-terminus (KHARP-GFP-Cter) of PfEMP1 were transported into the infected erythrocyte (Fig. 4A). Similarly, GFP chimeras containing the PfEMP1 C-terminus and the N-termini of rifin (Rifin-GFP-Cter), stevor (Stevor-GFP-Cter), and the predicted *P. berghei* exported protein (Pb-GFP-Cter) were transported into the erythrocyte (Fig. 4B). Thus, the N-termini of KAHRP, rifin, and stevor are functional in *P. berghei*, and the export process appears to be unaffected by the PfEMP1 C-terminus. In contrast, the PfEMP1 N-terminus alone or together with its C-terminus does not mediate export in *P. berghei*.

### Export of GFP to the erythrocyte surface

**Figure 5:**
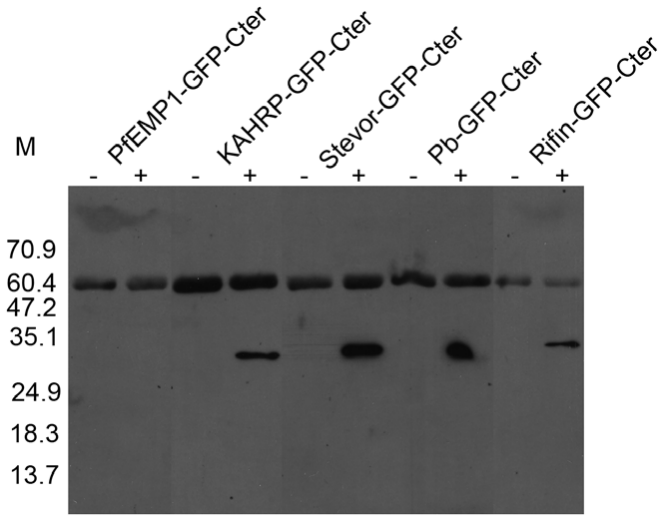
Export of GFP to the erythrocyte surface. Live trophozoite-infected erythrocytes expressing GFP reporter proteins with the PfEMP1 C-terminus at the carboxy terminus and the N-terminus of PfEMP1 (PfEMP1-GFP-Cter), KAHRP (KAHRP-GFP-Cter), stevor (Stevor-GFP-Cter), a predicted *P. berghei* exported protein (Pb-GFP-Cter), or rifin (Rifin-GFP-Cter) were incubated with (+) or without (−) TEV protease. Digestion samples were centrifuged, and supernatants were immunoprecipitated with protein-A agarose-coupled rabbit anti-GFP antibodies. The immunoprecipitates were processed for western blotting. The predicted sizes of full-length proteins in each case are >85 kDa and of the TEV cleaved products ≈30 kDa (36 kDa for PfEMP1-GFP-Cter). The presence of signal at ≈30 kDa in protease-containing samples indicates export of GFP to the surface of infected erythrocytes. The larger band (≈60 kDa) is likely due to cross reactivity of secondary antibodies with the heavy chain of rabbit anti-GFP antibodies used for immunoprecipitation. No larger signals indicating the presence of unprocessed proteins were seen.

Parasite-encoded proteins that are present on the surface of infected erythrocytes, notably PfEMP1family proteins, play key roles in immune evasion and pathogenesis [3], [28]. However, in vivo studies of PfEMP1-associated virulence have not been possible for several reasons, including the lack of easily available animal models. Identification of export elements mediating transport of PfEMP1 to the surface of *P. berghei*-infected erythrocytes may allow studies on PfEMP1-associated virulence in mice. Therefore, parasites expressing GFP chimeras of the PfEMP1 C-terminus and N-termini of KAHRP (KAHRP-GFP-Cter), rifin (Rifin-GFP-Cter), stevor (Stevor-GFP-Cter), the predicted *P. berghei* exported protein (Pb-GFP-Cter), and PfEMP1 (PfEMP1-GFP-Cter) were analyzed for the presence of GFP on the erythrocyte surface. These chimeras contained a TEV protease recognition site at the junction of GFP and the PfEMP1 C-terminus. As live cells are not permeable to TEV protease, treatment of live parasites with TEV protease would cleave GFP only if it is present on the surface of infected erythrocytes. Parasites expressing the GFP chimeras were treated with TEV protease, reaction supernatants were immunoprecipitated, and precipitated proteins were detected using anti-GFP antibodies. Expected sizes of products were detected in immunoprepitates from protease-treated KAHRP-GFP-Cter, Rifin-GFP-Cter, Stevor-GFP-Cter, and Pb-GFP-Cter, but not from PfEMP1-GFP-Cter and untreated parasites (Fig. 5). These data indicate that GFP is present on the surface of KAHRP-GFP-Cter, Rifin-GFP-Cter, Stevor-GFP-Cter, and Pb-GFP-Cter infected erythrocytes, and thus these N-termini together with the C-terminus of PfEMP1 mediate expression of PfEMP1 on the surface of *P. berghei*-infected erythrocytes.

## Discussion

The majority of known and predicted plasmodial exported proteins contain one or more transmembrane domains in their C-termini and one of three types of N-terminus domain architectures. The most common architecture, referred to here as Type 1, includes a hydrophobic/signal sequence preceding the PEXEL/VTS motif (KAHRP, rifin, stevor and the *P. berghei* putative exported protein (PB402722.00.0)). In Type 2 exported proteins, such as bir and SBP1, N-termini do not contain the PEXEL/VTS motif. Type 3 architecture is seen in PfEMP1 proteins, which lack a signal/hydrophobic sequence before the PEXEL/VTS motif. To determine if export of these three types of proteins utilizes a common or multiple mechanisms in plasmodia and to develop an efficient experimental system for studying protein export, we investigated export of GFP fused with the N- and C-terminus regions of proteins representing each type of domain architecture in the rodent parasite *P. berghei*. Of note, *P. berghei* allows much easier and more efficient transfection than does the human pathogen *P. falciparum*. Considering export signals from the N-terminus, Type 1 and Type 2 N-termini exported GFP into erythrocytes. However, the Type 3 N-terminus did not export GFP. Considering signals from the C-terminus, the C-terminus of rifin prevented GFP export by Type 1 N-termini, whereas the C-terminus of PfEMP1 allowed GFP export of the same N-termini to the surface of infected erythrocytes. These results suggest that the majority of exported plasmodial proteins, which have a Type 1 N-terminus, utilize a common export pathway, but that PfEMP1 export differs from this common pathway. Additionally, these results suggest that, while N-terminal signals principally direct export from the parasite, the final destination of exported proteins is guided by the C-terminus.

Export of GFP into *P. berghei*-infected erythrocytes by Type 1 N-termini of both *P. falciparum* and *P. berghei* exported proteins suggests the presence of a common export pathway in Plasmodia. Our results are consistent with a recently published study demonstrating export of GFP in *P. berghei*-infected erythrocytes by the N-terminus of *P. falciparum* histidine rich protein II, which has a Type 1 N terminus [27]. Although it has been suggested that bir proteins are also exported, the localization of these proteins in the infected erythrocyte has not previously been shown [12]. Our data showing export of GFP by a bir protein indicates that, as predicted, bir proteins are exported into the infected erythrocyte. Furthermore, export of GFP by a bir protein, which lacks a PEXEL/VTS motif, indicates that a PEXEL/VTS-independent mode of export is present in *P. berghei*. Although it is yet to be determined if bir or other Type 2 proteins also contain a signature export sequence analogous to the PEXEL/VTS motif in Type 1 proteins, recently published studies of the Type 2 proteins SBP1 and ring-exported protein-2 (REX2) revealed that 30 and 10 amino acid stretches of the N-termini of SBP1 and REX2, respectively, together with their C-terminus transmembrane and cytosolic domains are required for export in *P. falciparum* [8], [22]. The N-termini of SBP1 and REX2 do not appear to have a common motif, but both have a net negative charge; this feature has been proposed as a requirement for the export of SBP1 [22]. Interestingly, the first 40 amino acids of the N-terminus of the bir protein studied herein also has a net negative charge, suggesting that PEXEL/VTS-independent export employs a common mechanism in malaria parasites. The presence of a common export mechanism in malaria parasites is also supported by conservation of the components of a recently discovered transport complex in the parasitophorous vacuole membrane (PVM) of *P. falciparum*, which has been proposed to be required for protein export [20]. However, further studies are needed to determine if the PEXEL/VTS-independent export mechanism in *P. berghei* is similar to that present in *P. falciparum*.

PfEMP1 export is of particular interest because it is commonly associated with the virulence of *P. falciparum*. An in vivo system to study PfEMP1-associated pathogenesis is not available. The identification and functional validation of PfEMP1 export elements in *P. berghei* may lead to a system for expression of PfEMP1 on the surface of *P. berghei*-infected erythrocytes, which may be used for in vivo studies of cytoadherence. We, therefore, investigated transport of GFP chimeras containing N- and C-terminus regions of PfEMP1 in *P. berghei*. The N-terminus of PfEMP1 alone and together with its transmembrane domain or C-terminus did not export GFP in *P. berghei*-infected erythrocytes, suggesting that PfEMP1 export employs a mechanism different from that of Type 1 exported proteins. This mechanism may be unique to *P. falciparum*, which expresses a family of ∼60 PfEMP1 proteins [29]. The PfEMP1 used in this study has been localized to the surface of *P. falciparum*-infected erythrocytes, and has a well defined PEXEL/VTS motif (RNILE) [30]; N-termini of other PfEMP1s containing analogous sequences have been shown to export GFP in *P. falciparum*-infected erythrocytes [13], [14], [23]. Interestingly, another study found that a PEXEL/VTS sequence present further downstream in the first Duffy Binding Like (DBL) domain was required for export of a PfEMP1 into *P. falciparum*-infected erythrocytes [14]. As large proteins like PfEMP1 may have several PEXEL/VTS like motifs, it has not been definitively shown if PEXEL/VTS alone or other motifs are required for export of PfEMP1. Additionally, as PfEMP1 export has been shown to require several accessory proteins, including SBP1, the absence of SBP1 and some other proteins in *P. berghei* could explain the inability of the PfEMP1 regions to mediate export of GFP. Furthermore, knock out of SBP1 blocked export of PfEMP1 but not export of proteins with Type 1 and Type 2 architectures [10], [11]. These results further support the presence of a specific export mechanism for PfEMP1 in *P. falciparum*.

The majority of exported plasmodial proteins have one or more transmembrane domains in their C-termini, but the roles of these domains in the export process are not yet known. Considering that C-termini of proteins are often involved in targeting and anchoring at the target site, export of GFP fused to the rifin and PfEMP1 C-termini was investigated. The C-terminus of rifin, but not that of PfEMP1, prevented GFP export by Type 1 N-termini, including the N-terminus of the same rifin protein. This result was surprising, because the C-terminus of a different rifin protein did not prevent GFP export by its N-terminus in another study in *P. falciparum* [13]. Interestingly, a recent study demonstrated that there are two types of rifins: A and B [31], [32]. The A-type rifins are exported into the infected erythrocyte whereas B-type rifins are not exported [31]. The rifin studied herein is B-type, whereas the rifin that did not prevent GFP export in *P. falciparum* was A-type [13]. Thus, inhibition of GFP export by the C-terminus of rifin in our study is in agreement with the intraparasitic localization of B-type rifins in *P. falciparum*, and this result suggests that C-termini of exported proteins determine their destination. This study further suggests that the PEXEL/VTS motif transports proteins across the PVM and that the C-terminus then plays a key role in transport to the export destination. A more detailed study, including exported proteins with known destinations, is required to better understand the role of the C-terminus in determining the destination of export.

We demonstrated that GFP chimeras with Type 1 N-termini and the PfEMP1 C-terminus were exported to the surface of infected erythrocytes. This result provides proof of concept toward generation of transgenic *P. berghei* parasites expressing defined regions of PfEMP1 as a tool for in vivo studies of PfEMP1-associated pathogenesis. In fact, a similar reporter protein of GFP fused with the N-terminus of KAHRP and the C-terminus of PfEMP1 has been shown to export GFP to the surface of *P. falciparum*-infected erythrocytes [23]. Thus, this result suggests that even though PfEMP1 export is only present in *P. falciparum*, Type 1 N-termini with appropriate C-terminus regions may be used to transport proteins to desired locations in *P. berghei*.

In summary, we have characterized diverse signals for the export of proteins by *P. berghei*. Export was mediated in *P. berghei* by N-termini with a signal/hydrophobic sequence followed by the PEXEL/VTS motif (Type 1) and a bir protein that lacks a PEXEL/VTS motif (Type 2), but not by the N-terminus of PfEMP1, which lacks a signal/hydrophobic sequence before its PEXEL/VTS motif (Type 3). Thus, multiple export mechanisms appear to be present in malaria parasites. While the majority of proteins with a signal/hydrophobic sequence preceding the PEXEL/VTS motif may utilize a common export mechanism, the export of PfEMP1 appears to employ an alternative mechanism, which may be unique to *P. falciparum*. In addition, some proteins are exported without a PEXEL/VTS motif. However, it is possible that these three types of exported proteins utilize different pathways only for initial aspects of export with convergence to a common pathway. Functional validation of common putative export domains of malaria parasites in *P. berghei* provides an alternative and simpler system compared to *P. falciparum* to investigate export mechanisms. In addition, our data demonstrating export of GFP to the surface of *P. berghei*-infected erythrocytes may pave the way for generation of an expression system to display desired proteins, particularly defined cytoadherence regions of PfEMP1, on the surface of *P. berghei*-infected erythrocytes. This system might be used for in vivo studies of cytoadherence mediated by different PfEMP1 molecules.

## Materials and Methods

### Plasmid construction

We constructed the pSTCII transfection plasmid to express GFP reporter proteins in *P. berghei*. The pSTCII-GFP plasmid expresses GFP under the control of the upstream untranslated region of the *P. yoelii* chloroquine resistance transporter (PyCRT 5′) gene and downstream untranslated region of the *P. vivax* actin (PvAc 3′) gene. It has a pGEM backbone for replication in *E. coli*, and encodes the pyrimethamine resistant *Toxoplasma gondii* dihydrofolate reductase-thymidylate synthase gene (TgDHFR-TS) under the control of the *P. yoelii* α tubulin gene (PyαTb 5′) upstream untranslated region and the *P. vivax* α tubulin (PvαTb 3′) downstream untranslated region to allow selection of transfected parasites in *P. berghei*. The multiple cloning site contains Bgl II-Kpn I sites, a penta-alanine linker (Ala_5_), GFP followed by a penta-glycine (Gly_5_) linker, a *Tobacco etch* virus (TEV) protease cleavage site, and Bam HI-Xho I sites. To construct pSTCII-GFP, PyCRT 5′ (1.245 kb, primers: 5PyCRTF/5PyCRTR) and PyαTb 5′ (1.203 kb, primers: 5Py aTb1F/5PyaTb1R) were amplified from *P. yoelii* genomic DNA; PvAc 3′ (0.551 kb) (primers: 3UT Act F1/3UT ActR1) and PvαTb 3′ (0.566 kb) (primers: 3UT TbF1/3UT TbR1) were amplified from *P. vivax* genomic DNA; and the left and right flanks of the *P. berghei* C-small subunit ribosomal RNA (SSU) were amplified from *P. berghei* genomic DNA (primers: CSSUF/CSSULR; right flank: CSSURF/CSSURR) (see Table S1 for all primer sequences). The CSSULR and CSSURF primers were complementary (3′ of the left flank and 5′ of the right flank were overlapping), which allowed PCR-mediated recombination of left and right flanks with CSSUF and CSSURR primers to produce the CSSUL/R fragment (1.035 kb) containing central Avr II and Pac I sites. To mutate internal Age I, Xho I, and Bgl II sites of the pyrimethamine resistant *Toxoplasma gondii* dihydrofolate reductase-thymidylate synthase gene (TgDHFR-TS, 1.846 kb), the ORF was amplified in three fragments (TgDTA: F1/R1; TgDTB: F2/R2; F3/R3) using Phusion DNA polymerase and primers containing one base silent mutations corresponding to the restriction enzyme sites. The 5′ and 3′ ends of TgDTB were overlapping with the 3′ end of TgDTA and the 5′ end of TgDTC, respectively. These fragments were recombined into the mutated TgDHFR-TS gene (TgDTsm) with F1 and R3 primers using PCR. 3′-A overhangs were added to TgDHFR-TS using Taq DNA polymerase and deoxynucleotides at 70°C for 30 min, the tailed-product was cloned into the pGEM-T vector (Promega) to construct pGT-TgDTsm, and the product was sequenced to confirm desired sequence. The GFP coding sequence was amplified from the pSSPF2/HspGFP plasmid [33] using a mixture of Taq and Vent DNA polymerases with forward (GFPbsc F) and reverse (GFPbsc R) primers, to produce GFPbsc. 3′-A overhangs were added to GFPbsc using Taq DNA polymerase, the tailed-product was cloned into the pGEM-T vector (Promega) to construct pGT-GFPbsc, and the product was sequenced to confirm desired sequence. The pGEM backbone was excised from pHTK [34] by digesting with Nar I- Not I, and pGEM and the components described above were assembled into the pSTCII-GFP plasmid through several cloning and subcloning steps.

### Reporter constructs

Primers corresponding to N-termini of PfEMP1 (PFD1235w; 92 amino acids), KAHRP (PFB0100c; 70 amino acids), a predicted *P. berghei* exported protein (PB402722.00.0; Pb, 71 amino acids) and a bir protein (PB200037.00.0; FBir, flag tag +294 amino acid bir sequence) were used to PCR amplify the corresponding sequences from *P. falciparum* genomic DNA or cDNA or *P. berghei* genomic DNA using Phusion DNA polymerase (New England Biolabs). 5′- and 3′-A overhangs were added to these fragments, and they were cloned into the pGEM-T vector, sequenced, and excised with Bgl II-Kpn I. The Bgl II-Kpn I digested N-termini were cloned into similarly digested pSTCII-GFP to construct pSTCII-PfEMP1-GFP, pSTCII- KAHRP-GFP, pSTCII- Pb-GFP, and pSTCII-FBir-GFP plasmids. Primers used were: PfEMP1: NTSF/NTSR; KAHRP: K70F/K70R; Pb: PBpredF/PBpredR; FBir: BirF1/BirR1).

To construct pSTCII- PfEMP1-GFP-TM and pSTCII- KAHRP-GFP-TM plasmids, a 66 amino acid coding sequence of PfEMP1 (PFD1235w), including the 24 amino acid transmembrane region, was amplified from *P. falciparum* cDNA (TMATSF/TMATSR2). 5′- and 3′-A overhangs were added, and fragments were cloned into pGEM-T, sequenced, excised with Bam HI-Xho I, and cloned into similarly digested pSTCII- PfEMP1-GFP and pSTCII- KAHRP-GFP plasmids to construct the pSTCII- PfEMP1-GFP-TM and pSTCII- KAHRP-GFP-TM plasmids, respectively. The 454 amino acid C-terminus region (Cter) of PfEMP1 (PFD1235w) containing the transmembrane and cytosolic domains of PfEMP1 was amplified from *P. falciparum* cDNA (TMATSF/TMATSR1), mutated to remove the internal Kpn I site without changing the amino acid sequence, tailed with A-overhangs, cloned into pGEM-T, and sequenced. The Cter fragment was excised with BamH I-Xho I, and cloned into similarly digested pSTCII-PfEMP1-GFP and pSTCII- KAHRP-GFP to obtain pSTCII-PfEMP1-GFP-Cter and pSTCII- KAHRP-GFP-Cter, respectively.

To construct reporter plasmids containing N-termini of a rifin (PFA0745w), a stevor (PFC1105w), and a predicted *P. berghei* exported protein (PB402722.00.0, Pb), corresponding regions of these genes were amplified from *P. falciparum* cDNA or *P. berghei* genomic DNA using Phusion DNA polymerase and primers (Rifin: 51 aa, RifF/RifR; Stevor: 63 aa, SteF/SteR; Pb, 71 amino acids, PBpredF/PBpredR). These PCR products were tailed with A-overhangs as described above, cloned into pGEM-T, and sequenced. These fragments were excised with Bgl II-Kpn I and cloned into similarly digested pSTCII-PfEMP1-GFP-Cter replacing the Cter, and yielding pSTCII-rifin-GFP-Cter, pSTCII-stevor-GFP-Cter, and pSTCII-Pb-GFP-Cter plasmids.

The C-terminus regions of rifin were amplified from *P. falciparum* cDNA using Phusion DNA polymerase and primers (RCter, 78 aa: TMrifF/TMrifR; R_52-336_, 285 amino acids: TMRifF1/TMrifR). The pSTCII- PfEMP1-GFP-TM, pSTCII- KAHRP-GFP-TM, and pSTCII-rifin-GFP-Cter plasmids were digested with Bam HI-Xho I to excise the PfEMP1 transmembrane and C-terminus regions, and the plasmid backbones were ligated with a similarly digested RCter fragment to obtain pSTCII- PfEMP1-GFP- RCter, pSTCII- KAHRP-GFP- RCter, and pSTCII-rifin-GFP- RCter, respectively. Similarly, pSTCII-rifin-GFP-Cter was digested with Bam HI-Xho I to excise the PfEMP1 Cter, and the plasmid backbone was ligated with similarly digested R_52-336_ to obtain pSTCII-rifin-GFP- R_52-336_ plasmid. For the pSTCII-Rifin-GFP-Bir plasmid, bir protein coding sequence was amplified from *P. berghei* genomic DNA using Phusion DNA polymerase and primers BirF2/BirR2; the PCR fragment was digested with Bam HI-Xho I, and it was cloned into the plasmid backbone of similarly digested pSTCII-rifin-GFP-Cter replacing the PfEMP1 Cter.

### Parasite culture and isolation of genomic DNA and RNA

Studies with experimental animals were approved by the University of California, San Francisco Animal Care and Use Committee, and followed guidelines of this committee and the United States Department of Agriculture. *P. falciparum* 3D7 strain was cultured in RPMI1640 supplemented with 10% human serum [35]. Parasite-infected erythrocytes were harvested at 10–15% parasitemia. After centrifugation, the supernatant was aspirated off, and the pellet was treated with 0.1% saponin for 5 min in ice to lyse erythrocytes, and then centrifuged at 10000 rpm for 5 min. The supernatant was discarded, the pellet was washed twice with cold PBS to remove erythrocyte membranes, and parasites were recovered by centrifugation at 10000 rpm for 5 min. The supernatant was discarded, and the parasite pellet was processed for genomic DNA and RNA isolation using the PUREGENE™ DNA isolation kit (Gentra SYSTEMS) and Trizol method (Invitrogen), respectively. cDNA was produced from 5–10 µg genomic DNA-free RNA using the SuperScript First-Strand Synthesis System for RT-PCR (Invitrogen).

*P. berghei* ANKA strain (MRA-311) was obtained from Malaria Research and Reagent Reference Resource Center (MR4). A frozen stock was thawed at room temperature, injected into two 7–10 week old female Balb/c mice intraperitoneally with a 22G needle, and infection was monitored from day 3 onwards by making smears from tail vein snips. At ≈20% parasitemia, mice were euthanized and blood was collected by cardiac puncture in 10 ml Alsevier's solution. Blood was passed through the LeukoLOK filter (Ambion) to remove leukocytes and centrifuged at 2000 rpm for 5 min, supernatant was aspirated off, and the pellet containing parasite-infected red blood cells was processed for transfection or isolation of genomic DNA. For genomic DNA isolation, blood was processed as described above.

### Transfection

Transfection experiments were performed according to published protocols using the Nucleofector Device II and the human T-Cell Nucleofector kit (Amaxa) [36]. Briefly, the erythrocyte pellet containing *P. berghei*-infected cells was suspended in RPMI1640 (supplemented with 20% heat-inactivated fetal bovine serum) at 0.5–1% haematocrit in a sterile 250 ml plastic Erlenmeyer flask (Corning). The culture was gassed (5% CO_2_, 2% O_2_, and 93% N_2_) and incubated at 37°C at 85 rpm until the majority of parasites matured to schizonts. To purify schizont stage parasites, the parasite suspension was layered under a 55% Nycodenz gradient (Invitrogen) and centrifuged at 1000 rpm for 10 min in a swing bucket centrifuge without using brakes; interphase and top layers containing schizonts and free merozoites were collected and centrifuged at 3000 rpm for 5 min. The supernatant was discarded and the parasite pellet was washed twice with PBS, and parasites were used for 4–5 transfection experiments. For each experiment, parasites were mixed with 100 µl Nucleofector solution containing 5–10 µg transfection construct and electroporated using the U33 program of the Nucleofector Device II. The electroporated sample was immediately injected into the tail vein of a Balb/c mouse using a 30G needle. For selection of transfected parasites, mice were given pyrimethamine intraperitoneally (1.25 mg/100 µl of 50% DMSO/day) or in drinking water (70 mg/l H_2_O, pH 5.0) 24 hour post-electroporation and thereafter. Infection was monitored by making smears from tail snips every other day beginning on day 5. When parasitemia was >5%, mice were euthanized, and blood was collected as described above. 100–150 µl of the infected blood (diluted with PBS to 1% parasitemia) was injected intraperitonially into a naïve mouse for second round selection with pyrimethamine in drinking water. The remainder of the blood was centrifuged at 2000 rpm for 5 min, supernatant was aspirated off, and pellet was suspended in 2X volume of glycerolyte and stored at −80°C overnight followed by long term storage in liquid N_2_.

### Imaging of live parasites

5–10 µl blood was collected in 1 ml Alseviers' solution from the tail vein of a second round-selection mouse, the blood was centrifuged at 2000 rpm for 5 min, the supernatant was aspirated off, and the pellet was suspended in 3 ml RPMI1640 (supplemented with 20% FBS), and transferred to a 50 ml sterile tube. The tube was gassed and incubated at 37°C for 8–10 hrs to obtain trophozoite and schizont stages. 1 ml of the culture was transferred to a 1.5 ml microfuge tube, the tube was centrifuged at 14000 rpm for 10 seconds, the supernatant was aspirated off, and the pellet was washed twice with PBS and suspended in 1 ml PBS. Hoechst dye was added to the parasite suspension (1 µg/ml), and a 300–500 µl suspension was layered on a wet poly L-lysine coated glass slide (Sigma). The slide was incubated at room temperature for 10–15 min in a wet chamber (covered with aluminium foil), and unattached cells were washed off gently with PBS. A 20×40 mm glass coverslip (Sigma) was placed over the cells, and parasites were imaged under a 100 × objective lens using a Zeiss Axioplan epifluorescence microscope. Images were captured using a Hamamatsu CCD camera, and processed using Openlab software (Improvision).

### Protease digestion, immunoprecipitation, and immunoblotting

To determine surface localization of GFP, second round-selection parasites (expressing KHARP-GFP-Cter, Rifin-GFP-Cter, Stevor-GFP-Cter, Pb-GFP-Cter, and PfEMP1-GFP-Cter) were collected by cardiac puncture of euthanized mice as described above. Blood was passed through the LeukoLOK filter (Ambion) to remove leukocytes and centrifuged at 2000 rpm for 5 min, supernatant was aspirated off, and the pellet containing parasite-infected red blood cells was suspended in RPMI 1640 (supplemented with 20% heat inactivated fetal bovine serum) at 0.5–1% haematocrit in a sterile 250 ml plastic Erlenmeyer flask (Corning). The culture was gassed (5% CO_2_, 2% O_2_, and 93% N_2_) and incubated at 37°C at 85 rpm for 6–8 hours to obtain trophozoites. The culture was then centrifuged at 2000 rpm for 5 min; the supernatant was aspirated off and the pellet (300–380 µl packed cell volume of red blood cells with 8–10% parasitemia) was suspended in PBS and divided into two equal aliquots. The aliquots were centrifuged at 2000 rpm for 5 min, supernatants were aspirated off, and each pellet was suspended in 500 µl RPMI1640 containing 0.5 mM EDTA, 1 mM DTT, 0.1% BSA. 1.5 µl (15 U) of TEV protease (Invitrogen) was added to one aliquot, and an equal volume of the TEV protease storage buffer (50 mM Tris-HCl, pH 7.5, 1 mM EDTA, 5 mM DTT, 50% glycerol, 0.1% Triton X-100 (final Triton X-100 concentration 0.0003%)) was added to another aliquot. Both samples were incubated at room temperature for 90 min with gentle shaking, and then centrifuged at 14000 rpm for 2 min. The supernatants were transferred to 1.5 ml microfuge tubes. The volume of each supernatant was adjusted to 1 ml with PBS, including 30 µl of protein-A-agarose slurry (Pharmacia), 5 µl rabbit anti-GFP antibodies (Invitrogen), and 100 µl 10% BSA, and then incubated at 4°C overnight with gentle shaking. Each supernatant was passed through a spin column on a 2 ml microfuge tube; the flowthrough was discarded and the protein-A-agarose resin with immunoprecipate was washed twice with A1 buffer (50 mM Tris-Cl, pH 7.5, 150 mM NaCl, 1 mM EDTA, 0.1% NP40). The column containing the resin was placed on a 1.5 ml microfuge tube, 40 µl 1 x SDS-PAGE sample buffer was added directly over the resin, the sample was incubated at 95–100°C for 5 min, and it was then centrifuged at 14000 rpm for 2 min. Column flow throughs (≈30 µl) containing immunoprecipitates were run on a 12% SDS-PAGE gel and transferred onto the nitrocellulose membrane. The membrane was blocked with 3% nonfat dry milk in PBS-0.05% Tween-20, incubated with mouse anti-GFP antibodies (Invitrogen, Cat No. A1121) at 1/1000 dilution in the blocking buffer for 1 hr at room temperature, and then incubated with HRP-conjugated goat light chain-specific anti-mouse IgG (1/5000 in blocking buffer) for 1 hr at room temperature. The signal was developed with the SuperSignal Western Blotting kit (Pierce) on X-ray film (GE Healthcare).

## Supporting Information

Table S1 Primers used in the study.
(0.05 MB DOC)
