# Supplementary material for: Functional Evaluation of Plasmodium Export Signals in Plasmodium berghei Suggests Multiple Modes of Protein Export
Source: PLoS One. 2010 Apr 19;5(4):e10227. doi: 10.1371/journal.pone.0010227 (PMC2856681; doi:10.1371/journal.pone.0010227)
Supplement: Table S1 — Primers used in the study. (0.05 MB DOC) [file pone.0010227.s001.doc]

**Table S1.** Primers used in the study.

| **Primer** | **Sequence** |
| --- | --- |
| 5PyCRTF | TAT*ACcgGt*TTTACAAAATACTCCATCTCTCTT |
| 5PyCRTR | TACTGTCAT*agatct*TATTAATACACTGATTTG |
| 5Py aTb1F | TATA*ACCggT*GAAAAGCCCTAAATGCAAAACAT |
| 5PyaTb1R | ACTTCTCTCAT*ggatcc*TTTTACTTGTATATTAT |
| 3UT Act F1 | TGA*ctcgag*GGTGGGAGAAATTGACGAA |
| 3UT ActR1 | CACttAAttAACGCAGGCCTCCCTGGAAAA |
| 3UT TbF1 | AAGCGGATTATTAG*aaGctt*CGTGGCGTGA |
| 3UT TbR1 | TTG*cCtAGG*TGGGCATGTGTACATATATATAT |
| CSSUF | act*gcgGCcgC*ctcattaaaacagtta |
| CSSU LR | tttaatatacgctat*CCTagG*tTTaattaAcgcggctgct |
| CSSU RF | agcagccgcg*TtaattAA*aCctAGGatagcgtatattaaa |
| CSSU RR | actactcGtttaaaCaagatagttttatttat |
| F1 | atc*ggatcc*atgcataagccggtgtgt |
| R1 | cttccttctcttctcaagaaccacaaagtc |
| F2 | gactttgtggttcttgagaagagaaggaag |
| R2 | attcttgtcccagattttcacgcccttctc |
| F3 | gagaagggcgtgaaaatctgggacaagaat |
| R3 | acg*aagctt*gctagacagccatctccat |
| NTSF | ATAAGATCTCAAAATGGGGAATGCATCATCATCA |
| NTS R | AGGggtaccATTTCTATCTTCTGCGTTACGA |
| K70F | TAAagatctcaaaATGAAAAGTTTTAAGAACAAAAATACT |
| K70R | GTGggtaccTTGATGGTGATGGTGGTGATGGTGT |
| PBpredF | ataagatctcaaaATGGTATTCAAAATAAATGATTTTCT |
| PBpredR | TTGggtaccTTGATTATTCAATACGCTTGATT |
| BirF1 | CAAAATGGATTACAAGGATGACGACGATAAGagatctAACGTTTCTTTAgtaataatactat |
| BirR1 | TTAggtaccTTTTTTTAGCTTTTCTCTTAAATG |
| TMATSF | ACAggatccGAATCCTTATCACCTTCAGAGTCA |
| TMATSR2 | aaactcgagttaGCCTTTATGGATGTCAAGTACA |
| TMATSR1 | aaactcgagTTATATATTCCATACATCCGATATAGGA |
| RifF | AATagatctCAAAATGAAACTGCACTACACTAA |
| RifR | CTCggtaccATCATTATTATAATTTGTCGATTGT |
| SteF | AAA AGA TCT CAA AAT GAA GAT GTA TAA CCT TAA AAT GT |
| SteR | TCTggtaccGTCATTATGATAATGCGGATTAT |
| TMrifF | GTCggatccTCAGATGCCAAAAAAGCTGCT |
| TMrifR | tatctcgagTTATTGATTTAATAATTCTGTGTATTGGGCT |
| TMRifF1 | GATggatccGAGGATATCAAATCAGTGAAGGAA |
| BirF2 | ATGggatccAACGTTTCTTTAgtaataatacta (Bam HI-site replaced the starting codon ATG of the wild type gene) |
| BirR2 | tctctcgagTTATTTTTTTAGCTTTTCTCTTAAATG |
| GFPbscF | ata*AGATCT***CAAA**ATG*GGTACC*gcagccgcagcagctAGTAAAGGAGAAGAACTTTTCACT (Bgl II/Kpn I sites are italicized, Kozak sequence is in bold, and penta-alanine linker is underlined) |
| GFPbscR | ttt*CTCGAG***TTA***GGATCC*ctggaagtacagattttctcctccaccgcctccTTTGTATAGTTCATCCATGCCA (Bam HI-Xho I sites are italicized, stop codon is in bold, TEV protease site is double underlined, and glycine linker is single underlined) |
